# Notes on a Case of Alveolar Abscess, Complicated with Repeated and Profuse Hæmorrhage and Fracture of Inferior Maxilla

**Published:** 1891-09

**Authors:** Herbert R. Bowtell


ARTICLE VII.

### NOTES ON A CASE OF ALVEOLAR ABSCESS, COMPLICATED WITH REPEATED AND PROFUSE HÆMORRHAGE AND FRACTURE OF INFERIOR MAXILLA.

BY HERBERT R. BOWTELL, L. D. S., ENG.

J. H., Aet 35, was admitted into Charing Cross Hospital on August 11th, 1891, under the care of Mr. Stanley Boyd.

*History*:—On Friday, July 24th, patient received a blow on the right side of the jaw from a fist.

He came to the hospital on the following day, and was seen by the House Surgeon.

On examination, there was found on the right side of the lower jaw, a swelling of considerable size, which was extremely tender and painful. No crepitus or other signs of fracture could be detected, but the right lower wisdom was in a carious condition, and pus welled up between the alveolus and neck of this tooth in considerable quantity.

An antiseptic mouth-wash was prescribed, and patient advised to have his tooth extracted. When seen at the out-patients on the following day, it was found that the offending organ had been fractured, and an unsuccessful attempt made to remove the roots; and also that the swelling was a little smaller in size, but still painful, and pus readily obtainable.

Fluctuation could be felt inside the mouth, in the sulcus, between the cheek and alveolus.

The abscess was incised, and pus escaped in fair quantity.

When patient was again seen, August 11th, it was thought desirable to admit him, notwithstanding that the swelling was slightly smaller.

*Condition on Admission*:—The swelling over the right postero external portion of the lower jaw was considerable, and the cutaneous area red and œdematous, deep fluctuation being obtainable, pus was still welling up from the site of the fractured tooth.

The swelling was attached to the jaw, but no bare bone could be felt through an incision inside the oral cavity.

The patient having been placed under gas and æther, I attempted (at the request of the House Surgeon) the removal of the retained roots with the elevator, but without success, a small portion of the alveolus being removed at the time.

I next incised the abscess, keeping close to the alveolar margin.

Following this operation, a considerable amount of hæmorrhage occurred; the patient losing some 50 ozs. of blood before the bleeding could be controlled, which was eventually done with the assistance of ice bags and digital pressure.

This having been done, compressions was kept up for three hours by a cork covered with lint soaked in solution of tannin, and secured in position by means of a four-tailed bandage.

On August 18th, about a week after the foregoing operation, a pulsating swelling was discernible in the sulcus which shortly afterwards burst, being accompanied with profuse hæmorrhage, to the extent of 63 ozs., some shreddy pus being discharged at the same time. After this the bleeding ceased for a day.

On August 21st, owing to a severe recurrence of hæmorrhage, Mr. Boyd (after failing to secure the bleeding point, which seemed to be on the detached cheek, inside the mouth) made an incision of about 2 ins. in length along the lower border of the jaw, exposed the facial artery, and ligatured it, with the result that the bleeding from the mouth ceased.

The original wound was next plugged with iodoform gauze.

Whilst using the Mason's gag during the operation, the jaw became fractured at the site of the lesion, the fracture extending entirely through the body of the jaw, crepitus being easily obtained.

The wound having been sutured and dressed, the jaw was secured by a four-tailed bandage.

The temperature afterwards rose to 102° F., the patient being very anæmic and weak from loss of blood.

The mouth was washed out with boroglyceride, and the patient placed on a low diet.

On August 25th, the plug of iodoform gauze was removed, and the wound inside the mouth dusted with iodoform.

About midnight, the bleeding having recommenced, the patient was placed under chloroform.

Mr. Boyd then divided the lip in the mid-line, and reflected the cheek from the jaw, using and extending the incision which had been made for ligature of the facial artery.

The fracture produced at the former operation was then clearly visible. It extended in an oblique direction downwards and backwards, immediately in front of the angle of the jaw.

There was no displacement.

Above this a portion of the alveolus had been removed, and the abscess cavity exposed. By means of bone forceps the outer plate of bone was excised, and the inferior dental canal laid open.

The abscess cavity was next sharp-spooned and the retained roots removed.

On examination the inferior dental nerve and artery were found to be divided. Free bleeding had ceased, but it seemed probable that the inferior dental was the source of it.

A plug of catgut was rammed into both distal and proximal ends of the canal, and the hæmorrhage arrested.

The wound was powdered with iodoform, and the flap brought together with silver supporting sutures and a continuous horse-hair stitch. A sal-alembroth dressing was next applied and kept in position by a bandage.

Since this operation there has been no further hæmorrhage.

Remarks by Mr. Boyd:—

To the surgeon the interest of this case lies in the repeated hæmorrhage and their treatment. From Mr. Bowtell's account it seemed highly probable that the facial had been wounded in opening the alveolar abscess; and this view seemed to be supported by the formation of an arterial hæmatoma between the cheek and the jaw, the wound into the abscess cavity having healed to some extent so that blood was prevented from at once escaping externally.

On August 21st I was in the hospital when this hæmatoma burst, and on running to the patient found blood literally streaming from his mouth. I at once concluded that only the facial could yield such a stream, and when I at first got a good view of the cavity inside the cheek I saw blood issuing in a thickish column apparently from the cheek. I enlarged the wound in the mouth and tried to grasp the vessel with forceps; but after a few vain attempts, the bleeding was reduced to a general oozing (owing to the depression of the heart by the chloroform ?) and by no sponging or scraping of the surfaces could I make it bleed again. Under these circumstances I determined to tie the facial where it crosses the jaw—within half an inch of the bleeding spot. I was afraid to leave the patient open to the risk of fresh bleeding (he had lost at least 120 ozs. of blood in ten days) and I felt that if ligature at a distance from the bleeding spot could be relied upon at all this was the very case for it. On August 25th I was sent for at midnight, bleeding having recurred as freely as ever; many ounces of blood were lost before my house surgeon could reach the patient and plug the cavity in the mouth with his finger. The facial wound was healing nicely: we had not now to deal with secondary hæmorrhage from that vessel. As usual, bleeding had practically ceased when I arrived. Feeling that I must do everything in my power to find and deal with the bleeding point, I raised a flap, as for excision of the right half of the body of the jaw, and could find no bleeding point in its deep surface, but slow oozing was going on from the cavity in the bone which was full of clot. This was cleared out with a sharp spoon after I had cut away the outer plate of the jaw. I then found the two ends of the divided inferior dental nerve, the ends of the artery having retracted within the dental canal. I imagine that the complete division of the nerve was effected by the spoon; I thought that possibly the blood had come from a lateral wound in the artery. Unfortunately, there is no proof that the inferior dental was the vessel wounded, were there such, the case would be a striking instance of the fallacy which underlies the treatment of hæmorrhage by ligature at a distance.

Altogether, this man must have lost 150 ozs. of blood between August 11th and 25th, yet his condition after the last bout and operation was not such as to make me really anxious about him.—*Dental Record.*

